# Direct Interaction and Functional Coupling between Human 5-HT_6_ Receptor and the Light Chain 1 Subunit of the Microtubule-Associated Protein 1B (MAP1B-LC1)

**DOI:** 10.1371/journal.pone.0091402

**Published:** 2014-03-10

**Authors:** Soon-Hee Kim, Dong Hyuk Kim, Kang Ho Lee, Sun-Kyoung Im, Eun-Mi Hur, Kwang Chul Chung, Hyewhon Rhim

**Affiliations:** 1 Center for Neuroscience, Brain Science Institute, Korea Institute of Science and Technology, Seoul, Korea; 2 Department of Neuroscience, University of Science and Technology, Daejeon, Korea; 3 Department of Systems Biology, College of Life Science and Biotechnology, Yonsei University, Seoul, Korea; Karolinska Institute, Sweden

## Abstract

Serotonin (5-HT) receptors of type 6 (5-HT_6_R) play important roles in mood, psychosis, and eating disorders. Recently, a growing number of studies support the use of 5-HT_6_R-targeting compounds as promising drug candidates for treating cognitive dysfunction associated with Alzheimer’s disease. However, the mechanistic linkage between 5-HT_6_R and such functions remains poorly understood. By using yeast two-hybrid, GST pull-down, and co-immunoprecipitation assays, here we show that human 5-HT_6_R interacts with the light chain 1 (LC1) subunit of MAP1B protein (MAP1B-LC1), a classical microtubule-associated protein highly expressed in the brain. Functionally, we have found that expression of MAP1B-LC1 regulates serotonin signaling in a receptor subtype-specific manner, specifically controlling the activities of 5-HT_6_R, but not those of 5-HT_4_R and 5-HT_7_R. In addition, we have demonstrated that MAP1B-LC1 increases the surface expression of 5-HT_6_R and decreases its endocytosis, suggesting that MAP1B-LC1 is involved in the desensitization and trafficking of 5-HT_6_R via a direct interaction. Together, we suggest that signal transduction pathways downstream of 5-HT_6_R are regulated by MAP1B, which might play a role in 5-HT_6_R-mediated signaling in the brain.

## Introduction

Serotonin (5-hydroxytryptamine, 5-HT) is an important neurotransmitter modulating emotion, cognition, sleep, circadian rhythm, and motor functions [1]. Among seven subfamilies of 5-HT receptors (5-HT_1−7_ receptors), 5-HT_6_R, along with 5-HT_4_ and 5-HT_7_ receptors, is a G-protein-coupled receptor (GPCR) positively coupled to adenylate cyclase via Gαs proteins [2]. 5-HT_6_R has been considered as a promising therapeutic target for the treatment of neurological diseases because it is exclusively expressed in brain and has no known isoforms [3]. Highest expression of 5-HT_6_R is found in the striatum, amygdala, nucleus accumbens, hippocampous, cortex, olfactory tubercle, thalamus, and hypothalamus in the brain [4]. As expected from distribution, previous studies suggest that 5-HT_6_R plays an important role in cognition, mood, psychosis, and eating disorder [3]–[7]. However, molecular mechanisms by which such functions relate to 5-HT_6_R signaling are poorly elucidated. To understand 5-HT_6_R signaling, we employed a yeast two-hybrid screening method on a human brain cDNA library with the intracellular domains of human 5-HT_6_R. We previously reported that Fyn, a member of the Src family of non-receptor protein-tyrosine kinase, and Jun activation domain-binding protein-1 (Jab1) interact with human 5-HT_6_R and play significant roles in 5-HT_6_R-mediated signaling in the central nervous system [8], [9]. In the present study, we report that microtubule-associated protein 1B (MAP1B) directly binds to human 5-HT_6_R and functionally modulates its activities.

The vertebrate MAP1 family of microtubule-associated proteins consists of three members, MAP1A, MAP1B, and MAP1S. MAP1B, perhaps the best characterized MAP1 family protein, is predominantly expressed in the developing brain and found at adult stages albeit at lower levels [10]. By controlling microtubule stability and dynamics, MAP1B plays an important role in a variety of cellular functions in the nervous system, ranging from intracellular trafficking to neuritogenesis and degeneration [11]–[13]. Heavy and light chains of all MAP1 proteins contain structurally and functionally conserved domains that mediate heavy chain-light chain interaction, microtubule binding, and the association with F-actin either by direct interaction or binding to actin-binding proteins [14]. Light chains (LC) generated by proteolytic cleavage of MAP1A and MAP1B are called LC2 and LC1, respectively [14]. In this paper, we have identified that MAP1B interacts with 5-HT_6_R via LC1 (MAP1B-LC1). We have also found that MAP1B-LC1 increases 5-HT_6_R activities by using an FDSS6000 system-based assay and probing changes in extracellular signal-regulated kinase 1/2 (ERK1/2) phosphorylation, well-known downstream signaling of 5-HT_6_R activation. Furthermore, we suggest regulation of surface expression and endocytosis of the 5-HT_6_R as an underlying mechanism for the MAP1B-LC1-mediated up-regulation of 5-HT_6_R signaling.

## Materials and Methods

### Yeast two-hybrid assay

Yeast two-hybrid assay was performed using the Matchmaker GAL4 two-hybrid system 3 (Clontech, Palo Alto, CA) as described previously [8]. The bait plasmid, pGBKT7/CT of 5-HT_6_R, and the prey plasmid, human brain cDNA library/pACT2, were transformed into yeast strain AH109 and Y187, respectively. After mating of two yeast clones with each other, the diploid colonies were plated on a nutritionally selective plate deficient in adenine, histidine, leucine, and tryptophan (-Ade, -His, -Leu, -Trp) to screen the library. False positives were eliminated using two reporters, ADE2 and HIS3, and MEL1-encoding β-galactosidase was assayed on 5-bromo-4-chloro-3-indolyl-α-D-galactopyranoside (X-α-gal) indicator plates. Doubly positive clones were isolated and characterized by DNA sequencing. β-Galactosidase activity for a yeast two-hybrid assay was measured using a β-galactosidase colony-lift filter assay in accordance with the manufacturer’s instructions (Clontech).

### Cell line culture and transfection

HEK293, HeLa, and SH-SY5Y cells were cultured in Dulbecco’s modified Eagle’s medium (DMEM) or DMEM: Nutrient Mixture F-12 (DMEM/F12 for SH-SY5Y) supplemented with 10% fetal bovine serum, 100 units/ml penicillin, and 100 µg/ml streptomycin at 37°C in a humidified atmosphere containing 5% CO_2_. HEK293 cells stably expressing the HA-tagged 5-HT_6_R (HEK293/HA-6R) and HeLa cells stably expressing the HA-tagged 5-HT_6_R (HeLa/HA-6R) were maintained with 400 µg/ml of G-418. For transient transfection, cells were transfected with each plasmid DNA using Lipofectamine PLUS reagent (Invitrogen, Calsbad, CA). After 24 h of transfection, the cells were prepared for further experiment.

### Primary culture of hippocampal neuron and transfection

All experiments involving animals were performed in accordance with the animal protocol approved by the Institutional Guidelines of Korea Institute of Science and Technology. Dissection and culture of hippocampal neurons were performed as described previously [15]. In brief, hippocampi from newborn mice (postnatal day 1) were collected in a Ca^2+^- and Mg^2+^-free HBSS solution and then digested with papain (Worthington) and DNAse I (Sigma) for 40 min at 37°C. Hippocampi were then washed two times with HBSS solution, followed by trituration. Dissociated cells were plated at a density of 100,000 cells/well into 24-well plates containing poly-D-lysine-coated glass coverslips. Neurons were cultured in Neurobasal media supplemented with B27 and 2 mM GlutaMAX (Invitrogen). After 9 days of culture, neurons were transfected using Lipofectamine 2000 (Invitrogen). At 24 hr after transfection, the cells were fixed for immunocytochemistry.

### GST pull-down assay

Plasmids inserted with glutathione S-transferase (GST) and GST-carboxyl-terminus (CT) of the 5-HT_6_R were transformed into *E.coli* BL21 (DE3). Their protein expressions were induced by adding 0.5 mM isopropyl 1-thio-β-D-galactopyranoside at 25°C during the midlog phase. The cells were harvested and lysed by sonication. All GST-tagged proteins (GST-4RCT, GST-6RCT, and GST-7BRCT) were immobilized on glutathione gel. Flag-LC1 gene was transfected into HEK293 cells, and cells were harvested and lysed in lysis buffer after 24 h transferction. HEK293 cell lysates containing Flag-LC1 protein were incubated with immobilized GST-tagged proteins. GST pull-down assay was performed using the Profound Pull-down GST Protein:Protein Interaction kit (Pierce, Rockford, IL). After three washes with lysis buffer, bound proteins were eluted by boiling for 10 min at 95°C in SDS sample buffer, followed by immunoblotting with anti-Flag (Cell Signaling Technology, Beverly, MA) and anti-GST (Novagen, Madison, WI) antibodies.

### Co-immunoprecipitation

Co-immunoprecipitation was performed as described previously [8], [9]. Briefly, cell and brain lysates were precleared with 50 µl of ImmunoPure immobilized protein G Plus (Pierce) and 2 µg of rabbit normal IgG for 1 h. Precleared lysates were incubated with 4 µg of anti-HA (Cell Signaling Technology) and anti-5-HT_6_R (GeneTex Inc., San Antonio, TX) antibodies overnight at 4°C. The lysates were then incubated with 50 µl of ImmunoPure immobilized Protein G Plus for 4 h at 4°C and were washed three times. Immune complex were eluted by boiling for 5 min at 95°C in SDS sample buffer, followed by immunoblotting.

### Immunoblotting

After 12% SDS-polyacrylamide gel electrophoresis, the proteins were transferred to nitrocellulose membrane (Millipore, Bedford, MA). The membranes were blocked with Tris-buffered saline containing 5% skim milk and 0.1% Tween-20 for 1 h at room temperature (RT). After blocking, the membranes were incubated with the respective primary antibodies (anti-Flag, anti-HA, anti-MAP1B, or anti-5-HT_6_R antibodies) overnight at 4°C. After three washes, the membranes were incubated with horseradish peroxidase (HRP)-conjugated secondary antibodies (Jackson ImmunoResearch, West Grove, PA) for 1 h at RT. The immune complexes were visualized with an ECL detection kit (Millipore).

### Immunocytochemistry

Immunocytochemistry was performed as previously described [16] with minor modifications. Cells were fixed with 4% paraformaldehyde at RT for 15 min. Fixed cells were washed three times with phosphate-buffered saline (PBS) and blocked in blocking solution (2% BSA, 0.1% Triton X-100, and 0.1% sodium azide in PBS). GFP-5-HT_6_R was labeled with chicken anti-GFP antibodies (Abcam, 1:1000), followed by labeling with anti-chicken Alexa Fluor 488 (Invitrogen). Endogenous MAP1B was stained with anti-MAP1B antibodies (Abcam, 1:200), followed by labeling with anti-mouse Alexa Fluor 568 (Invitrogen). All secondary antibodies (1:500) were incubated at RT for 1 hr. Cells were viewed with an inverted light microscope (Zeiss Axio observer Z1, Carl Zeiss MicroImaging, Inc) equipped with epifluorescence optics. Images were captured with a CCD camera controlled by Zen software (Carl Zeiss MicroImaging, Inc). A 20 X objective (LD Plan-Neofluar, NA 0.4) was used to record whole neurons. Images were cropped and/or enlarged to show localizations in sufficient detail.

### Assay of 5-HT_6_R activity using an FDSS6000 system

5-HT_6_R activity was measured using an FDSS6000 96-well fluorescence plate reader (Hamamatsu Photonics, Japan) as previously described [17]. Briefly, HEK293 cells were transiently transfected with Gα_15_ and 5-HT receptors (5-HT_4_R, 5-HT_6_R, or 5-HT_7B_R) using Lipofectamine Plus. After transfection, cells were seeded into 96-well black wall/clear bottom plates and cultured overnight. The cells were loaded with 5 µM Fluo-4/AM and 0.001% Pluronic F-127 (Molecular Probes, Eugene, OR) and incubated in an HEPES-buffered solution (150 mM NaCl, 5 mM KCl, 1 mM MgCl_2_, 10 mM HEPES, 10 mM glucose and 2 mM CaCl_2_, pH 7.4) for 1 h at 37°C. After three washes, 5-HT receptor activities were assayed with the FDSS6000 system. After determination of a short baseline, 10 µM of 5-HT was added to the cells, and the Ca^2+^ response was measured at 480 nm. All data were collected and analyzed using the FDSS6000 system and related software (Hamamatsu Photonics).

### cAMP accumulation assay for 5-HT_6_, 5-HT_4_, and 5-HT_7B_ receptors

To analyze cAMP levels, cAMP dynamic 2 HTRF kits (Cisbio, France) which provide homogeneous high-throughput assay were used. Cells incubated at 37°C in 5% CO_2_ and 95% air atmosphere were suspended in PBS containing 2 mM IBMX (3-isobutyl-1-methylxanthine) and stimulated by 10 µM 5-HT. After 30 min, cAMP labeled with the dye d2 and anti-cAMP antibodies labeled with cryptate were added into the cell plates. The plates were incubated at room temperature for 1 h. The fluorescence intensity of accumulated cAMP level was measured at 314 nm excitation, and 668 and 620 nm emission using Flexstation3 microplate reader (Molecular Devices, Downingtown, PA).

### Surface biotinylation assay

Surface biotinylation assay was performed using Pinpoint Cell Surface Protein Isolation kit (Pierce) as previously described [8]. Briefly, cells were washed with ice-cold PBS and incubated with 0.5 mg/ml of biotin solution in PBS for 30 min at 4°C. To quench the reaction, glycine was added into the cells to a final concentration of 100 mM, and cells were harvested and lysed in lysis buffer. The lysate was added to immobilized streptavidin bead and incubated for 2 h at 4°C. After three washes with lysis buffer, bound proteins were eluted by boiling for 5 min at 95°C in SDS sample buffer followed by immunoblotting with anti-HA, anti-Flag, and anti-β-tubulin (Cell signaling Technology) antibodies.

### Assessment of membrane/cytosolic distribution of 5-HT_6_R

Cellular distribution of 5-HT_6_R was examined by tracing the GFP that fused to N-terminal of 5-HT_6_R. HEK293 cells were transiently transfected with GFP-5-HT_6_R and Flag-LC1 (or Flag empty vector) genes, and plated and cultured on coverslip for 24 h. The cells were fixed with 4% paraformaldehyde in PBS for 20 min at RT. After wash with PBS, GFP expression in the cells was observed with fluorescence microscope (Olympus, Japan), and the distribution and the amount of fluorescence were analyzed with Meta-Morph imaging program (Molecular Devices, Downingtown, PA).

### Receptor endocytosis assay

ELISA-based receptor endocytosis assay was performed according to the method previously reported [18]. HeLa/HA-6R cells were transiently transfected with control Flag empty vector or Flag-tagged LC1. After 24 h transfection, the cells were treated with 100 µM 5-HT for 10 min, fixed, and blocked with 1% BSA and 1 mM CaCl_2_. The cells were incubated with anti-HA antibody (1∶1000) for 1 h and then HRP-conjugated secondary antibody (1∶2000) for 30 min at RT. After washes, the HRP substrate *o*-phenylenediamine dihydrochloride (Sigma-Aldrich) was added and incubated for 30 min in the dark. To stop the reaction, 3 N HCl was added, and absorbance was measured at 492 nm using VersaMax ELISA Microplate reader (Molecular Devices). Receptor endocytosis is expressed as % of receptors initially present at the membrane, and represents mean ± S.E. of at least five independent experiments carried out in triplicate.

### Statistical analysis

All experiments were independently repeated three times. The intensity of bands was measured using Image J software ((National Institute of Health, Bethesda, MD) and analyzed using the GraphPad Prism program (GraphPad Software Inc., San Diego, CA). All numeric values are represented as the mean ± S.E. The statistical significance of the data was determined using a Student’s unpaired *t* test or one-way analysis of variance (ANOVA) followed by Duncan’s tests. Significance was set at *p<*0.05, and the values of *P* for significant differences are indicated in the text and figure legends.

## Results

### The LC1 of MAP1B is responsible for 5-HT_6_R binding

**Figure 1:**
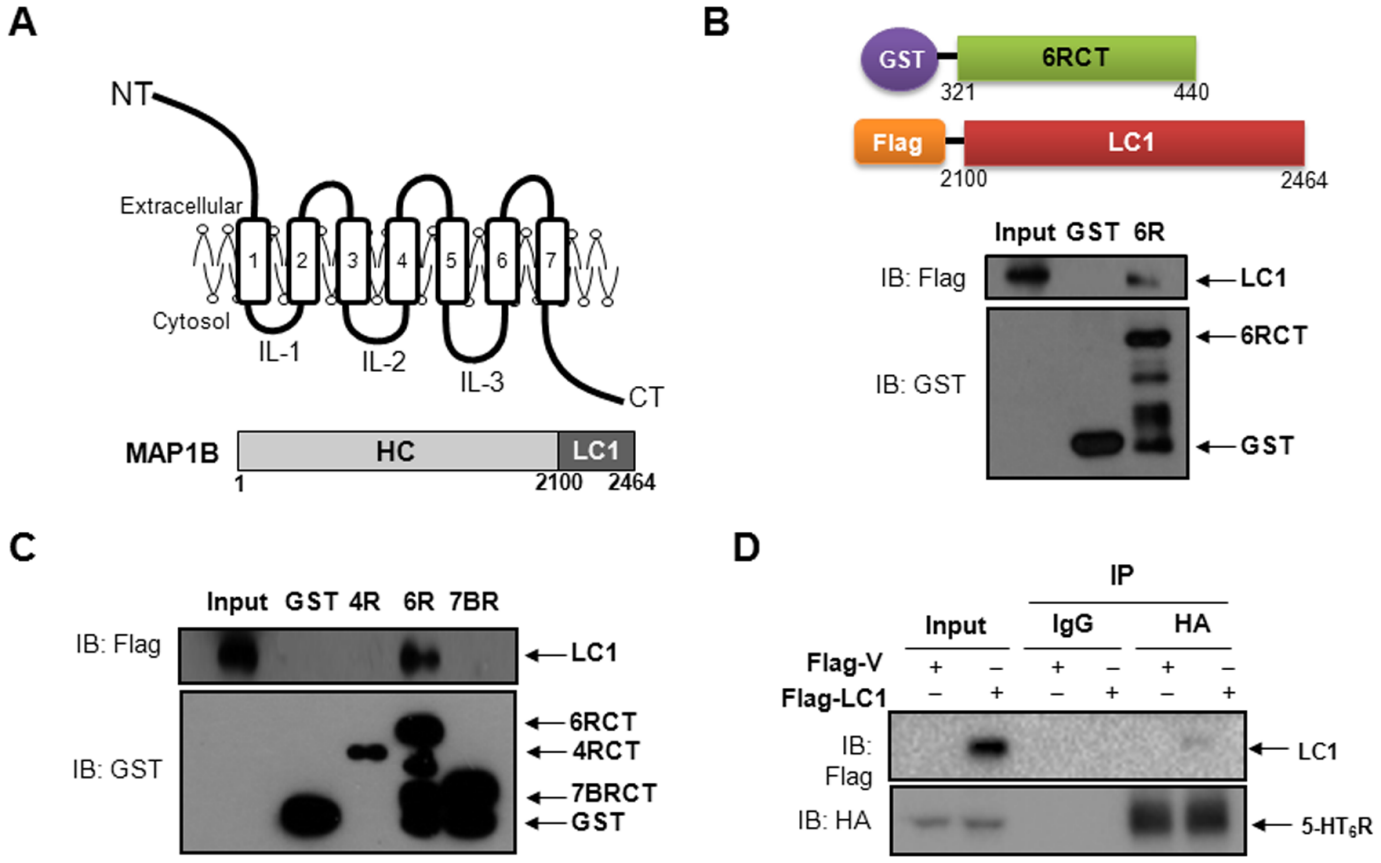
Interaction of MAP1B-LC1 with 5-HT_6_R by using GST pull-down and co-immunoprecipitation assays in HEK293/6R cells. (A) Schematic diagrams of the 5-HT_6_R and MAP1B. Numbers indicate amino acid positions for human MAP1B (according to database entry XM_005248507). (B) *Upper,* Schematic illustration of GST-fused CT of 5-HT_6_R and Flag-tagged LC1 of MAP1B; *lower*, direct binding between CT of 5-HT_6_R and MAP1B-LC1 using GST pull-down assay. (C) GST pull-down assays between MAP1B and CTs of 5-HT_4_, 5-HT_6_, or 5-HT_7B_ receptors (GST-4RCT, GST-6RCT, or GST-7BRCT). (D) *In vitro* interaction between Flag-LC1 and HA-5-HT_6_R was determined by immunoprecipitation with anti-HA antibodies in HEK293/HA-6R cells.

We previously demonstrated that the C-terminal (CT) region of human 5-HT_6_R interacts with Fyn tyrosine kinase and characterized the downstream signaling pathways of 5-HT_6_R activation regulated by Fyn [8]. We also reported an interaction between 5-HT_6_R and Jab1 and investigated how Jab1 modulates the membrane expression and activity of 5-HT_6_R [9]. Furthermore, we demonstrated that 5-HT_6_R and Jab1 play important roles under conditions of hypoxia *in vitro* and cerebral ischemia *in vivo*. In the present study, we have identified a new binding partner of 5-HT_6_R, MAP1B-LC1, by performing a yeast two-hybrid screening on a human brain cDNA library. We have found that MAP1B-LC1 also binds to the CT region of human 5-HT_6_R based on the yeast two-hybrid screening assay and confirmed the interaction by a GST pull-down assay. Fig. 1A shows schematic diagrams of the 5-HT_6_R and MAP1B. As shown in Fig. 1B, Flag-tagged LC1 specifically interacted with GST-6RCT. To investigate the selectivity of binding between the 5-HT_6_R and MAP1B, we examined whether MAP1B interacts with other serotonin receptors. Among several serotonin receptors, we tested 5-HT_4_R and 5-HT_7B_R which are coupled to Gαs as 5-HT_6_R is. To this end, we performed GST pull-down assays using intracellular CT regions of 5-HT_4_R (4RCT) and 5-HT_7B_R (7BRCT) as bait proteins. As shown in Fig. 1C, 4RCT and 7BRCT did not bind to MAP1B, while interaction between 6RCT and MAP1B was readily detected.

**Figure 2:**
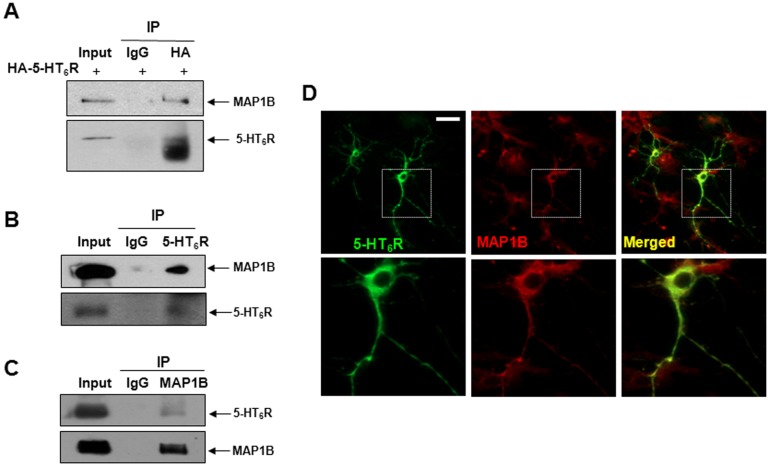
MAP1B directly interacts with 5-HT_6_R in neuroblastoma cells and the brain. (A) *In vitro* interaction between MAP1B and HA-5-HT_6_R was determined by immunoprecipitation by using anti-HA antibodies in SH-SY5Y cells. (B & C) *In vivo* interaction between 5-HT_6_R and MAP1B was examined by immunoprecipitation with anti-5-HT_6_R or anti-MAP1B antibodies in the fetal rat brain. (C) Colocalization of 5-HT_6_R-GFP and MAP1B in cultured mouse hippocampal neurons. Shown are representative images of hippocampal neurons stained for 5-HT_6_R-GFP (green) and endogenous MAP1B (red). Boxed areas are enlarged in lower panels. Bar, 20 µm.

To validate the interaction that we observed *in vitro* (through the yeast expression system and the GST pull-down assay), we examined their interaction in mammalian cell lines and rat brain by performing a co-immunoprecipitation assay. For this purpose, Flag-tagged LC1 was transiently transfected into HEK293 cells stably expressing the HA-tagged 5-HT_6_R (HEK293/HA-6R), and then cell lysates were subjected to immunoprecipitation with anti-HA antibodies, followed by immunoblotting with anti-Flag antibodies. As shown in Fig. 1D, Flag-LC1 was able to bind to HA-5-HT_6_R in HEK293 cells. We then tested the interaction in human neuroblastoma SH-SY5Y cells, which express MAP1B endogenously. SH-SY5Y cells were transiently transfected with HA-5-HT_6_R, immunoprecipitated with anti-HA antibodies, and subsequently immunoblotted with anti-MAP1B antibodies. As shown in Fig. 2A, HA-5-HT_6_R was able to bind to endogenous MAP1B protein in SH-SY5Y cells, whereas no signal was detected in immunoprecipitates using control IgG antibody.

We also detected the association between 5-HT_6_R and MAP1B in rat brain lysates. As shown in Fig. 2B, endogenous 5-HT_6_R selectively bound to endogenous MAP1B (third lane) in rat brain, whereas no signal was detected in immunoprecipitates using control IgG antibody (second lane). We confirmed the interaction by performing co-immunoprecipitation in a reverse manner, where anti-MAP1B antibodies were used for immunoprecipitation, followed by immunoblotting with 5-HT_6_R antibodies (Fig. 2C). We next examined the co-localization of 5-HT_6_R and MAP1B using double immunofluorescence staining methods. In cultured hippocampal neurons, we examined the co-localization of endogenous MAP1B and exogenously transfected GFP-5-HT_6_R. As illustrated in Fig. 2D, GFP-5-HT_6_R and MAP1B were present at the cell membrane and also in neurites. These results strongly demonstrate that MAP1B interacts with human 5-HT_6_R not only in mammalian cell lines, but also in native brain and hippocampal neurons.

### MAP1B-LC1 selectively modulates the 5-HT_6_R activity

**Figure 3:**
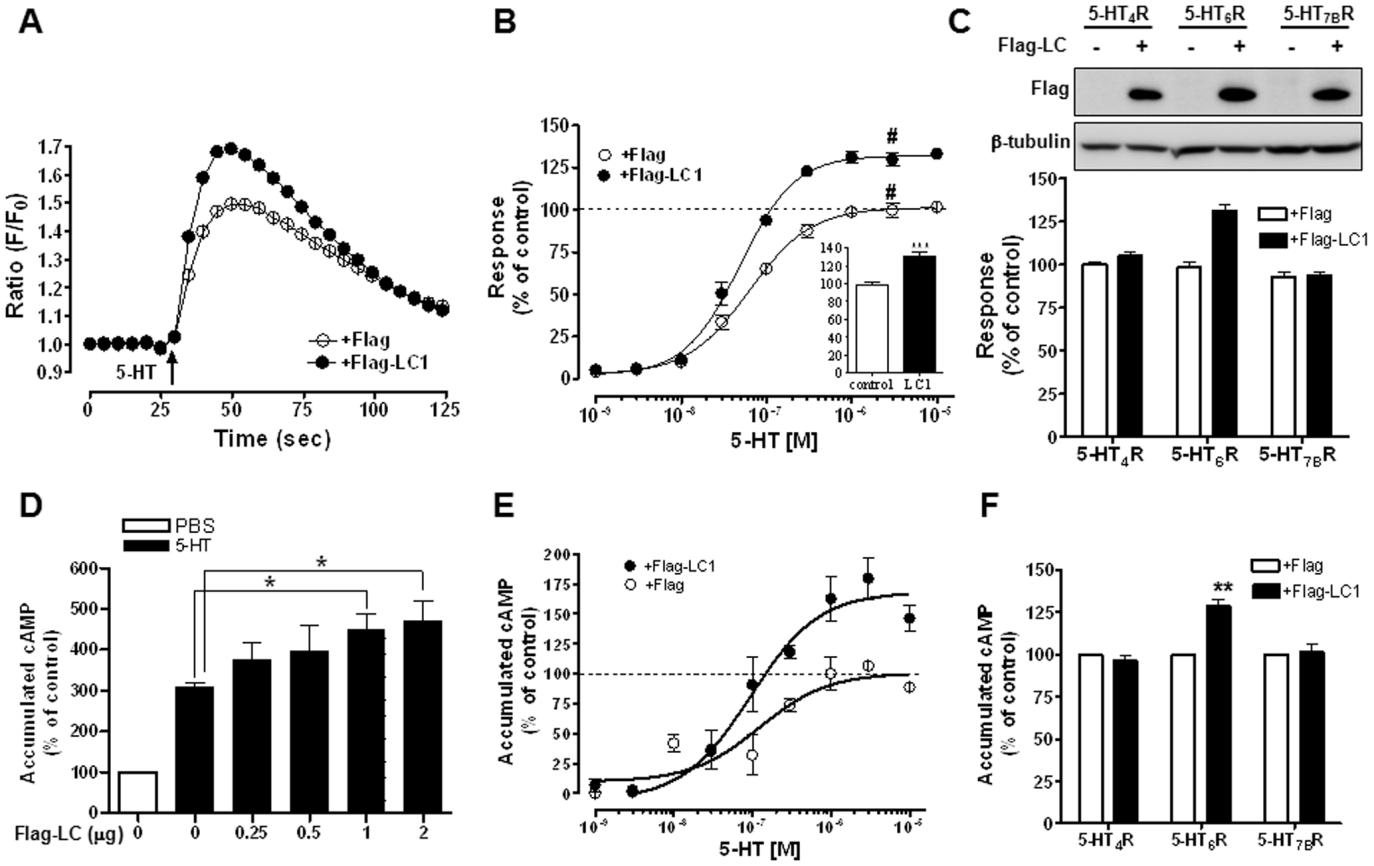
Expression of MAP1B-LC1 selectively regulates 5-HT_6_R activities. (A) HEK293 cells were transiently transfected with 5-HT_6_R and Gα_15_ in the presence of Flag-vector or Flag-LC1.At 24 h after transfection, Ca^2+^ responses induced by 10 µM of 5-HT were measured by using an FDSS6000 system, as described in Materials and Methods. *F* represents fluorescence intensity, and *F_0_* denotes initial fluorescence intensity at 480 nm. (B) Dose-dependency of 5-HT_6_R-mediated Ca^2+^ responses in the absence or presence of Flag-LC1. *Inset*, pooled results showing 5-HT-induced Ca^2+^ response measured at the indicated dose (#, presented as a percentage value of control). (C) Effect of LC1 overexpression on the activities of other Gαs-family 5-HT receptors. The 5-HT_4_R, 5-HT_6_R, or 5-HT_7B_R was transfected into HEK293 cells along with Flag-LC1, and intracellular Ca^2+^ responses were measured after 5-HT treatment. (D) Measurements of accumulated cAMP in response to 10 µM of 5-HT and increasing amounts of Flag-LC1 (0 – 2 µg). Reponses are compared to PBS-treated control condition. (E) Dose-response curve of 5-HT_6_R-mediated cAMP levels in the absence or presence of Flag-LC1. (F) Effects of LC1 on cAMP levels in response to activation of HT_4_R, 5-HT_6_R, or 5-HT_7B_R. *, *p<0.05*; **, *p<0.01*

Physical interaction between 5-HT_6_R and MAP1B via the LC1 subunit suggests that MAB1B-LC1 might affect 5-HT_6_R activity. To test this possibility, we applied our previously developed assay system to assess 5-HT_6_R activities [17], [19]. In this assay system, co-transfected Gα_15_ along with 5-HT_6_R facilitates the association of Gα_S_–coupled receptors with phospholipase C, and subsequently increases intracellular Ca^2+^ release, which can be detected by using an FDSS6000 96-well fluorescence plate reader. As shown in Fig. 3A, ectopic expression of Flag-LC1 significantly increased 5-HT-induced intracellular Ca^2+^ response in HEK293 cells that had been transiently transfected with 5-HT_6_R and Gα_15_. This LC1-mediated increase in 5-HT_6_R activity was observed over a wide range of 5-HT concentrations, detected from 1 nM to 10 µM (Fig. 3B). Concentration-response curves to 5-HT showed a significantly increased maximum response with LC1 expression (131.1±3.8% of control in the presence of Flag-LC1). However, there was no apparent change in 5-HT_6_R affinity. The calculated EC_50_ values were 62.3±11.2 nM and 43.6±6.1 nM in the absence and presence of Flag-LC1, respectively (*n* = 9, p = 0.38). To ensure that LC1-induced upregulation of 5-HT_6_R activity is specific to the 5-HT_6_R subtype, we examined whether overexpression of LC1 brings a change in the activities of 5-HT_4_R and 5-HT_7B_R. As shown in Fig. 3C, the expression of LC1 specifically increased 5-HT_6_R activity without changing 5-HT_4_R or 5-HT_7B_R activities. We confirmed equivalent expression levels of Flag-LC1 in the three conditions expressing different types of 5-HT receptors.

**Figure 4:**
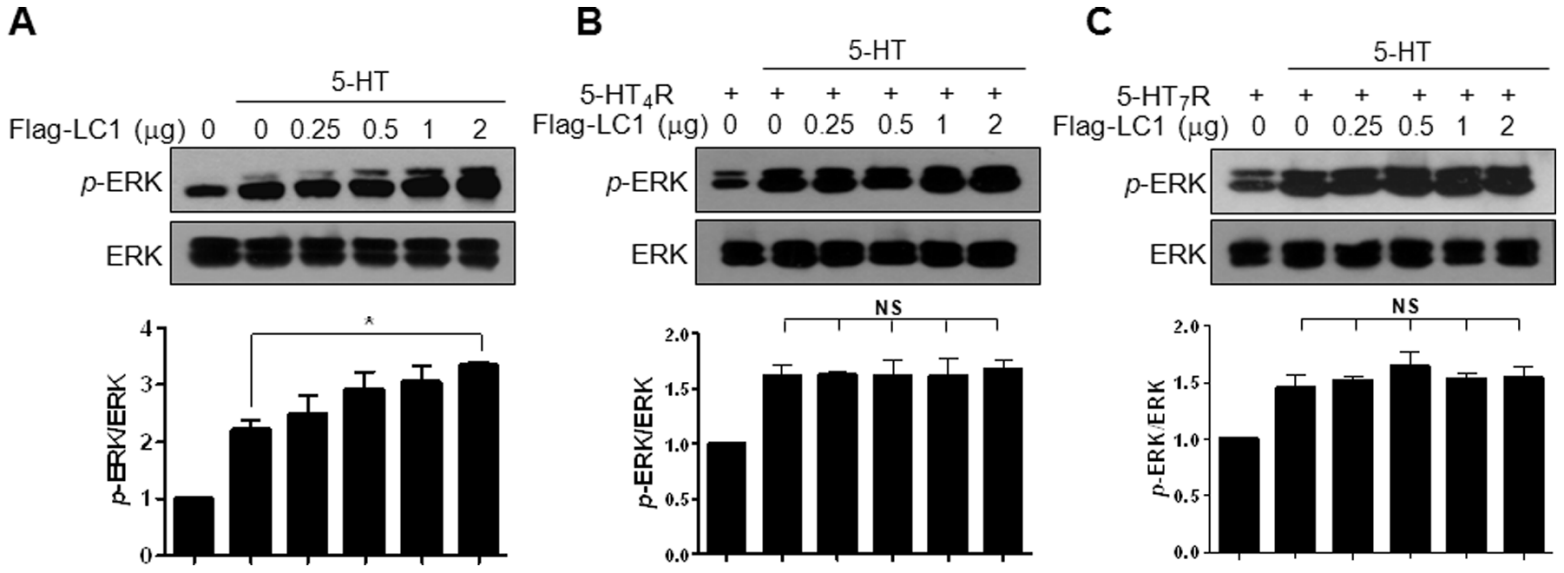
Effects of MAP1B-LC1 on the 5-HT receptor activities assayed using ERK1/2 activation. 5-HT-mediated ERK1/2 activation was measured in HeLa/HA-6R cells (A) and HeLa cells transiently transfected with HT_4_R (B) or 5-HT_7B_R (C). Flag-LC1 was transiently transfected into HeLa/HA-6R or naïve HeLa cells, and ERK1/2 phosphorylation induced by 5-HT (10 µM, 5 min) was measured at 24 h after transfection. NS, not significant.

Because 5-HT_6_R is positively coupled to adenylate cyclase via Gαs proteins, we examined the effect of MAP1B-LC1 expression on 5-HT-induced cAMP level. As indicated in Fig. 3D, overexpression of Flag-LC1 increased 5-HT-induced cAMP level in HEK293/HA-6R cells. Coexpression of Flag-LC1 with 5-HT_6_R enhanced 5-HT-elicited increase in cAMP level without changing 5-HT_6_R apparent affinity for 5-HT (Fig. 3E). The calculated EC_50_ values were 74.9±21.0 nM and 90.2±23.7 nM in the absence and presence of Flag-LC1, respectively (*n* = 3). This Flag-LC1-induced increase in cAMP level occurred exclusively in 5-HT_6_R-expressing but not 5-HT_4_R- or 5-HT_7B_R-expressing HEK293 cells (Fig. 3F). Recent studies including ours have reported that these Gαs family 5-HT receptors induce Ras-dependent activation of extracellular signal-regulated kinase 1/2 (ERK1/2) [8], [20]. We, therefore, confirmed the specific effects of LC1 on 5-HT_6_R by using ERK1/2 phosphorylation as a readout in other cell-types. We used HeLa/HA-6R cells for monitoring 5-HT_6_R activation, and naïve HeLa cells transiently expressing 5-HT_4_R or 5-HT_7B_R for measuring 5-HT_4_R or 5-HT_7_R activation. As shown in Fig. 4A, treatment with 10 µM 5-HT increased ERK1/2 phosphorylation in HeLa/HA-6R cells. This was further increased by the expression of increasing amounts of LC1. However, 5-HT-induced ERK1/2 phosphorylation was not affected in 5-HT_4_R- or 5-HT_7B_R-expressing HeLa cells despite that 5-HT-mediated ERK1/2 phosphorylation was evident in these cells (Figs. 4B and C). Taken together, these results suggest that MAP1B-LC1 specifically modulates 5-HT_6_R activity.

### MAP1B-LC1 modulates surface expression and endocytosis of the 5-HT_6_R

**Figure 5:**
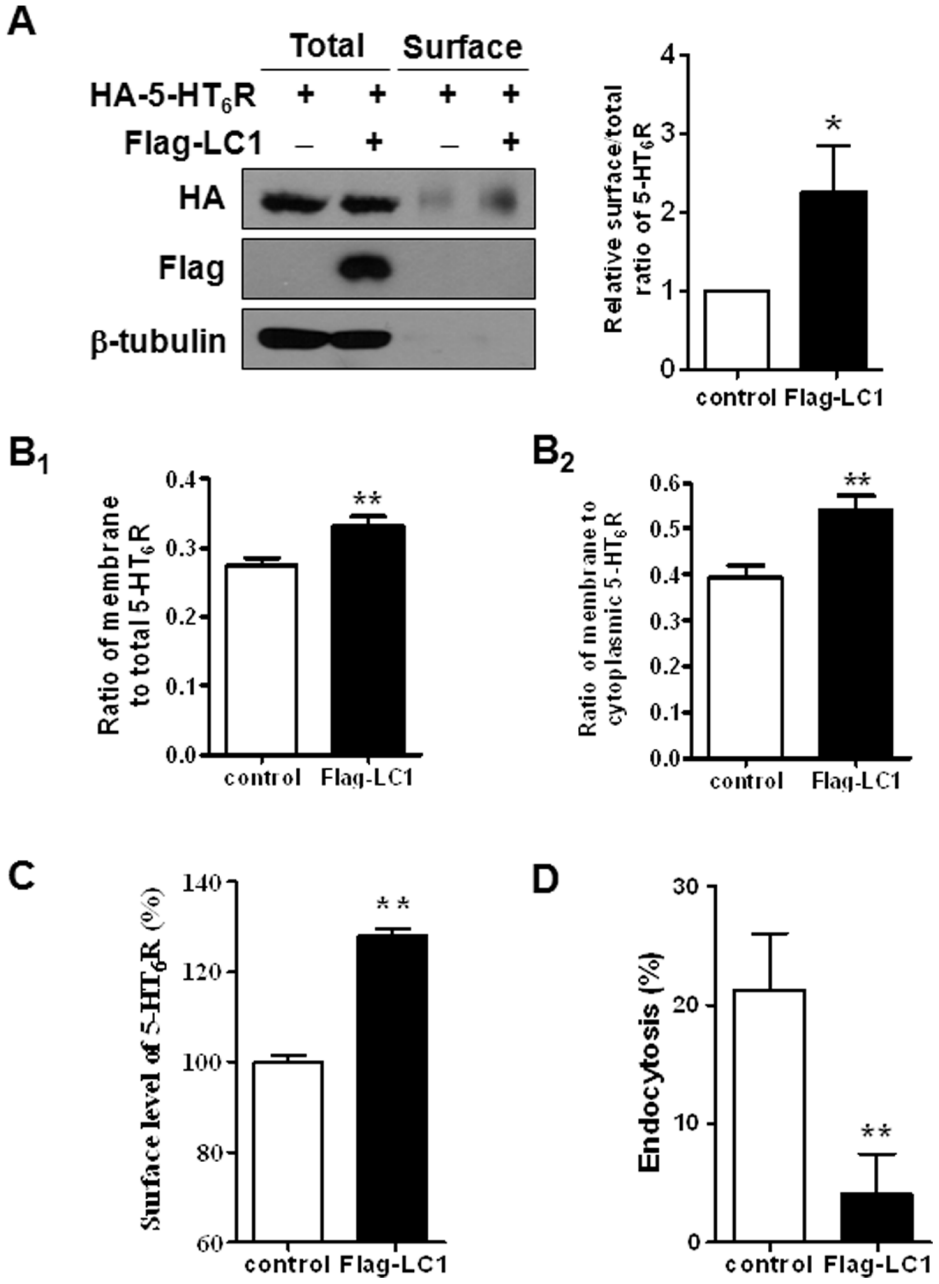
MAP1B-LC1 modulates the surface expression and agonist-induced endocytosis of 5-HT_6_R. (A) Effects of MAP1B-LC1 on the surface expression of 5-HT_6_R. Biotinylation experiments from HEK293/HA-6R cells showed that MAP1B-LC1 increased the amount of 5-HT_6_R at the cell surface, but total levels were unaffected. Data were normalized to the surface level of 5-HT_6_R in cells transfected with a control vector. (B) GFP-fused 5-HT_6_R was transfected into HEK293 cells in the absence (control) or presence of Flag-LC1. After 24 h, fluorescence intensity of GFP in the cell membrane and the cytoplasm, as well as total fluorescence levels were analyzed by using Metamorphor program. (C) HeLa/HA-6R cells were transiently transfected with a control or Flag-LC1. At 24 h after transfection, the amounts of HA-5-HT_6_R present on the cell surface were measured by performing ELISA with anti-HA antibodies. (D) Under the same condition as in (C), cells were treated with 100 µM of 5-HT for 10 min, and changes in surface 5-HT_6_Rs were quantified. Receptor endocytosis is expressed as a percentage value of surface receptors initially present at the membrane before 5-HT treatment. Data were obtained from six independent experiments carried out in triplicate.

We next examined how overexpression of LC1 increased 5-HT_6_R activity without changing the affinity for the receptor, as previously illustrated in Figs. 3B and E. We quantified the surface expression of 5-HT_6_R in the absence or presence of Flag-LC1 using surface biotinylation experiments. As shown in Fig. 5A, HEK293/HA-6R cells transfected with Flag-LC1 exhibited more 5-HT_6_R at the cell surface than cells transfected only with Flag empty vector (2.27±0.58 fold increase compared to control, *n* = 3, *p<*0.05). There was no difference in the level of total expression for 5-HT_6_R proteins. To confirm these results, we examined cellular distribution of 5-HT_6_R by tracing GFP fused to the N-terminal of 5-HT_6_R using immunofluorescence methods. At 24 h after transfection of HeLa cells with GFP-fused 5-HT_6_R and LC1, cellular distribution of 5-HT_6_R was examined. As shown in Fig. 5B_1_, the ratio of membrane to total 5-HT_6_R was significantly increased by the expression of Flag-LC1. The ratio of membrane to cytoplasmic 5-HT_6_R was also significantly enhanced in Flag-LC1-expressing cells as compared to control cells (0.41±0.03 vs. 0.54±0.03, *n* = 31, *p<*0.05, Fig. 5B_2_). We reaffirmed these results by using ELISA-based receptor endocytosis assay in HeLa/HA-6R cells. The increased surface level of 5-HT_6_R was again observed in LC1-expressed HeLa/HA-6R cells (Fig. 5C), supporting the results from biotinylation experiments in HEK293/HA-6R cells. Under the same condition, the cells were treated with 100 µM of 5-HT for 10 min, and the difference in the amount of surface 5-HT_6_R before and after 5-HT treatment was evaluated as endocytosis. Endocytosis of the 5-HT_6_R was significantly reduced from 21.3±4.8% (control) to 4.0±3.3% (with Flag-LC1) (Fig. 5D). Taken together, these results suggest that LC1 binding to 5-HT_6_R supports the existence of 5-HT_6_R in the membrane, which consequently increases its activity.

### Functional interaction between the 5-HT_6_R and MAP-LC1 using the Flag-CT and selective ligands of the 5-HT_6_R

**Figure 6:**
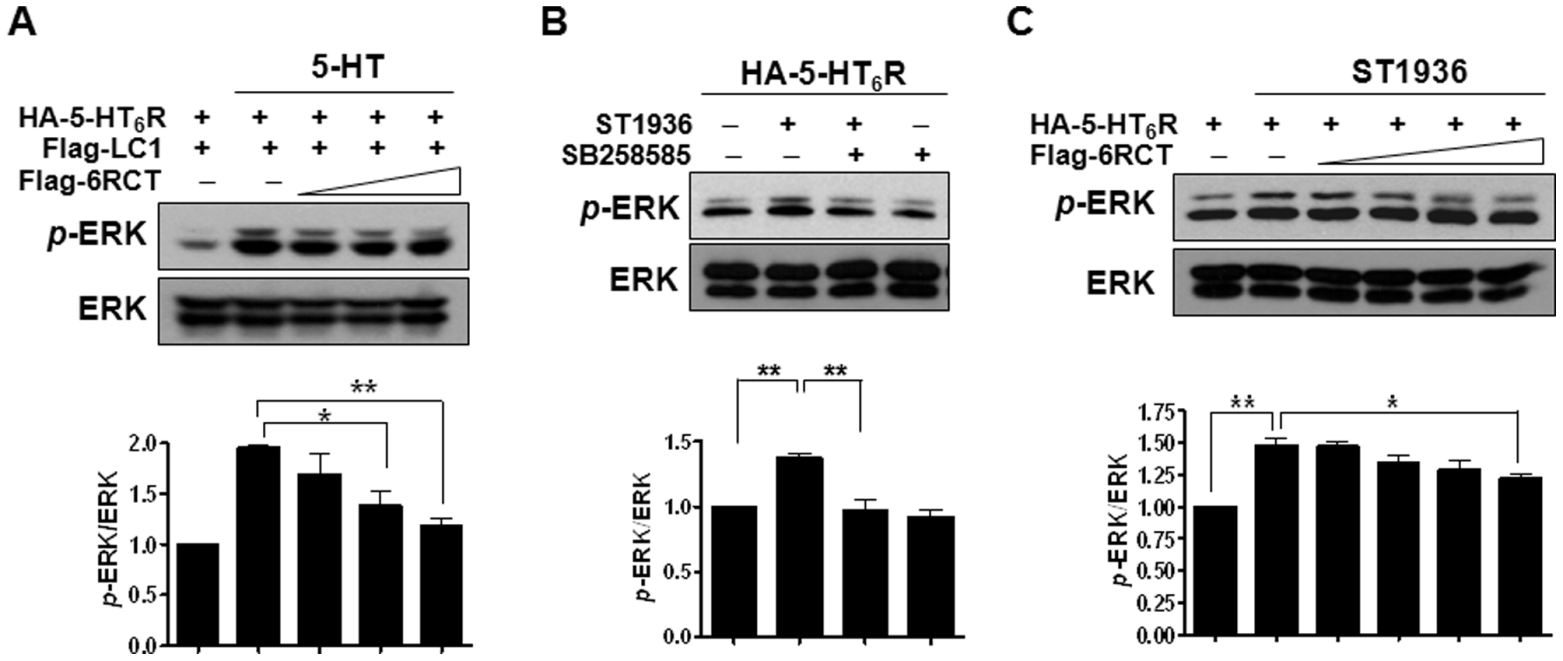
The effects of overexpression of the carboxyl terminus of 5-HT_6_R on ERK1/2 phosphorylation. (A) ERK1/2 phosphorylation was examined in HEK293/HA-6R cells transfected with Flag-LC1 (1.5 µg) and varying amounts of Flag-6RCT (0, 0.375, 0.75, or 1.5 µg). (B) ST1936 (the selective agonist of 5-HT_6_R)-mediated ERK1/2 phosphorylation in SH-SY5Y cells. ST1936 and SB258585, selective antagonists of 5-HT_6_R, were used at the concentration of 10 µM. (C) Effects of overexpression of Flag-6RCT on 5-HT_6_R-mediated ERK1/2 phosphorylation in SH-SY5Y cells transfected with HA-5-HT_6_R (1.5 µg) and varying amounts of Flag-6RCT (0, 0.375, 0.75, or 1.5 µg).

To further examine the functional modulation of 5-HT_6_R signaling by MAP1B-LC1, we interfered with the interaction by overexpressing the CT region (amino acid 321-440) of 5-HT_6_R, the binding site of MAP1B-LC1, in HEK293/HA-6R and SH-SY5Y cells. As shown in Fig. 6A, 5-HT-induced ERK1/2 phosphorylation was significantly reduced by the expression of Flag-tagged CT (Flag-6RCT) in HEK293/HA-6R cells. We next examined whether similar results can be obtained from SH-SY5Y cells endogenously expressing MAP1B. At 24 h after transfection of SH-SY5Y cells with either HA-5-HT_6_R alone or together with Flag-6RCT, we examined ERK1/2 phosphorylation upon treatment with a selective agonist or a selective antagonist of 5-HT_6_R, alone or in combination. To specifically activate the 5-HT_6_R type without stimulating other types of 5-HT receptors that might be expressed in this neuroblastoma cell line, we applied a recently developed selective agonist of 5-HT_6_R, ST1936 [21]. In SH-SY5Y cells, 10 µM of ST1936 significantly increased ERK1/2 phosphorylation, which was blocked by the treatment with 10 µM of SB258585, a selective antagonist of 5-HT_6_R (Fig. 6B). Treatment with SB258585 alone had no effect on ERK1/2 activation. When Flag-6RCT was overexpressed under these conditions, we observed that Flag-6RCT inhibited ERK1/2 phosphorylation induced by ST1936 (Fig. 6C). These results indicate that interaction of MAP1B-LC1 with the C-terminal tail region of 5-HT_6_R regulates the activation of ERK1/2.

## Discussion

Since the discovery of the human 5-HT_6_R by Kohen et al. [2], increasing numbers of selective and novel 5-HT_6_R ligands have been developed using high-throughput screening technologies [3], [16]. The synthesis of 5-HT_6_R ligands, especially 5-HT_6_R antagonists, has been very successful along with considerably fewer compounds claimed to be selective 5-HT_6_R agonists [6], [7]. Currently, consistent effects have been demonstrated with 5-HT_6_R antagonists in preclinical models of cognition, and the role of these receptors in depression and anxiety has also been postulated although the majority of 5-HT_6_R *in vivo* research has focused on their pro-cognitive effects. However, the preclinical results are somewhat equivocal since both blockade and stimulation of 5-HT_6_R produce pro-cognitive, antidepressant-like, or anti-anxiety-like effects [3]. The explanation for these paradoxical effects remains unclear. Therefore, it is essential to investigate molecular mechanisms by which 5-HT_6_R signaling is connected to such functions. Insights into how 5-HT_6_R signaling affects brain function can be provided by the identification of specific 5-HT_6_R-binding proteins along with the elucidation of how 5-HT_6_R activity or signaling is modulated by such interactions. For example, Meffre et al. [22] recently demonstrated physical interactions between 5-HT_6_R and several proteins involved in the mTOR pathway and identified a molecular substrate underlying modulation of cognition by receptor ligands.

In our previous studies [8], [9], two direct interacting proteins, Fyn and Jab1, have been identified. Stimulation of 5-HT_6_R induces ERK1/2 phosphorylation through Fyn, and binding with Fyn upregulates the surface expression of 5-HT_6_R as well as its activity [8]. In another study, we have shown that Jab1 binds to 5-HT_6_R, and upon 5-HT_6_R stimulation, Jab1 translocates from the cytoplasm to the nucleus, leading to the phosphorylation of c-Jun, which in turn enhances the association between Jab1 and c-Jun [9]. In the present study, we have found a third novel binding protein of 5-HT_6_R, MAP1B-LC1 and report three major findings on the physical and functional interaction between 5-HT_6_R and MAP1B-LC1.

Firstly, this is an original report demonstrating direct interaction between human 5-HT_6_R and the LC1 subunit of MAP1B protein. Formation of the 5-HT_6_R-MAP1B-LC1 complex was confirmed by using yeast two-hybrid, GST pull-down, and co-immunoprecipitation assays in two different human-derived cell lines as well as in native rodent brains and hippocampal neurons. We initially employed a yeast two-hybrid screening on the human brain cDNA library with cDNA fragments encoding the CT of 5-HT_6_Rs and found MAP1B-LC1 as a binding partner. This specific interaction was further confirmed by using a yeast two-hybrid assay (data not shown) and a GST pull-down assay. Furthermore, co-immunoprecipitation assays in SH-SY5Y neuroblastoma cells and the rat brain (Fig. 2) suggest full length of both 5-HT_6_Rs and MAP1B interact with each other in native tissues. In addition, immunocytochemisty assays demonstrated that 5-HT_6_R and MAP1B co-localized at the cell membrane and neural processes in cultured hippocampal neurons. Among the intracellular loops 1, 2, 3 (iL1, iL2, and iL3, schematic illustration provided in Fig. 1), and CT regions of the 5-HT_6_R, we used the CT region as a bait because the CT is a common binding region interacting with both Fyn and Jab1 and because physical interaction of this region with the two binding-proteins elicits important effects on HT_6_R signaling [8], [9]. Further investigation is necessary to examine if other regions in 5-HT_6_R (e.g. iL1, iL2, or iL3) bind to MAP1B, and subsequently regulate 5-HT_6_R function. Here we showed there is no direct interaction of MAP1B to intracellular CT regions of 5-HT_4_R and 5-HT_7B_R, but additional experiments are required to examine if other regions in 5-HT_4_R or 5-HT_7B_R interact with MAP1B.

Secondly, to the best of our knowledge, the present work is the first report to show modulation of 5-HT_6_R activities and downstream signaling by MAP1B-LC1. Modulation of 5-HT_6_R activities and downstream signaling was confirmed by monitoring various responses, including 5-HT_6_R-mediated Ca^2+^ increase, -cAMP accumulation, and ERK1/2 activation. Although MAP1B is known as an abundant neuronal cytoskeleton protein, MAP1B-LC1 specifically modulated the activation and signaling of the 5-HT_6_R type, while leaving those of other Gαs-coupled 5-HT receptors, such as 5-HT_4_R and 5-HT_7_R, intact. Through intensive investigation using multiple 5-HT_6_R activity assays, we demonstrated that the expression of Flag-LC1 significantly and consistently increased 5-HT_6_R activities. By contrast, the expression of Flag-LC1 had no effect on the activation or signaling of 5-HT_4_R or 5-HT_7_R.

Thirdly, we have investigated the mechanism by which MAB1B-LC1 controls 5-HT_6_R signaling and found that MAP1B-LC1 modulates the surface expression and endocytosis of 5-HT_6_R. Results from surface biotinylation, direct observation of GFP-fused receptors, and endocytosis assays consistently revealed that the expression of MAP1B-LC1 increased the surface level of 5-HT_6_R and decreased agonist-induced desensitization. Taken together, these results suggest that MAP1B-LC1 functions to retain the surface expression of 5-HT_6_R, and thereby prevents basal and agonist-induced receptor desensitization via a direct interaction, which provides a feasible explanation for the MAP1B-LC1-mediated upregulation of 5-HT_6_R signaling.

The MAP1B-LC1 binds to a variety of signaling molecules, including tumor suppressor p53 [23], RNA-binding protein HuD [24], sodium channels [25], and Rho GTPase regulatory protein, Tiam1 [26]. However, GPCRs are rarely reported as protein interaction partners of MAP1B-LC1. Moritz et al. [27] reported that metabotropic glutamate receptor 4 (mGluR4), a GPCR involved in the regulation of neurotransmitter release, bound to MAP1B, but the region in MAP1B mediating the association, and more importantly, the functional consequences of this interaction on mGluR4 signaling were not investigated. Here, we not only identify the subunit of MAP1B responsible for mediating the specific interaction with 5-HT_6_R, but also demonstrate functional consequences of this interaction. Interestingly, a previous report showed a direct interaction between MAP1B-LC1 and a ligand-gated ion-channel, 5-HT_3_R in HEK293 cells [10]. In contrast to our study, MAP1B-LC1 reduced the surface expression of 5-HT_3_R and accelerated 5-HT_3_R gating properties, such as desensitization kinetics. Whether the interaction occurs in neurons or the brain is still unknown, and the physiological significance of decreased surface expression of 5-HT_3_R by MAP1B-LC1 may need to be evaluated in neuronal cells. Nevertheless, these studies suggest that MAP1B-LC1 might play an important role in the regulation of 5-HT neurotranstamitter signaling by controlling surface expression and/or trafficking of receptors and channels.

One important question that should be addressed is how overexpression of the CT region of 5-HT_6_R affects 5-HT- or ST1936-mediated 5-HT_6_R activities as shown in Fig. 6. In our previous study, we used Fyn-SH3 domain, the site in Fyn that binds to 5-HT_6_R, to specifically interfere with the interaction between 5-HT_6_R and Fyn [8]. Overexpression of Fyn-SH3 domain decreased 5-HT-induced pY420 Fyn phosphorylation and ERK1/2 phosphorylation. In the present study, we interrupted the interaction between 5-HT_6_R and MAP1B by expressing the CT region of 5-HT_6_R, and we observed that overexpression of the CT region significantly inhibited 5-HT- and ST1936-mediated ERK1/2 phosphorylation both in HEK293 and SH-SY5Y cells. The effects of MAP1B-LC1 binding to 5-HT_6_R were quite similar to those of Fyn binding [8]. Fyn also functions to increase the surface expression of 5-HT_6_R and 5-HT_6_R-mediated signaling [8]. Given that both Fyn and MAP1B interact with the CT region in 5-HT_6_R, it is possible that the disruption of 5-HT_6_R signaling induced by overexpression of the CT region of 5-HT_6_R could result from interfering with the interaction of 5-HT_6_R with both Fyn and MAP1B. As the two interacting partners retain the surface expression of 5-HT_6_R, which subsequently augments 5-HT_6_R signaling, it is plausible to suggest that binding of Fyn to 5-HT_6_R affects the association between MAP1B and 5-HT_6_R. Similarly MAP1B associating with 5-HT_6_R might have an effect on Fyn-5-HT_6_R interaction, and subsequently modulate 5-HT_6_R-signaling. Previous studies have demonstrated that Fyn can directly phosphorylate a microtubule-associated protein, Tau, which interacts with the SH3 domain in Fyn [28], [29]. Therefore, it is tempting to speculate that Fyn and MAP1B work in concert to control microtubule dynamics in the vicinity of 5-HT_6_R, but further research is required to test this possibility.

In conclusion, our results suggest that MAP1B-LC1, an important cytoskeleton protein in the CNS, is involved in the desensitization and trafficking of 5-HT_6_R and consequently controls 5-HT_6_R-mediated signal transduction via a direct interaction.
